# High absorption shielding material of poly(phthalazinone etherketone)/multiwall carbon nanotube composite films with sandwich configurations†

**DOI:** 10.1039/c9ra02959a

**Published:** 2019-06-14

**Authors:** Yunping Hu, Ping Tang, Longwei Li, Junyu Yang, Xigao Jian, Yuezhen Bin

**Affiliations:** Department of Polymer Science and Engineering, Faculty of Chemical, Environmental and Biological Science and Technology, Dalian University of Technology No. 2 Linggong Rd Dalian 116024 P. R. China binyz@dlut.edu.cn +0411-84986093 +0411-84986093; Department of Chemical Engineering, University of Pittsburgh 4200 Fifth Avenue Pittsburgh PA 15260 USA

## Abstract

Sandwich structure can be induced to achieve excellent electromagnetic interference shielding effectiveness (EMI SE) as is well known. However, how to optimize the structure to achieve performance optimization is still a problem. Herein, a poly(phthalazinone etherketone)/multiwall carbon nanotube (PPEK/MWCNT) composite with homodisperse and high content (15 wt%) of MWCNT was prepared by a water induced phase separation process. A sandwich structure was designed by introducing a wave-transmitting layer between the PPEK/MWCNT composite films. The MWCNT loading of the shielding layer and the wave-transmitting layer thickness (*d*) were varied to systematically investigate their influence on shielding performance in the X-band (8.2–12.4 GHz). EMI SE shows strong *d* value dependence. When the shielding layer was fixed, EMI SE showed a trend of decreasing, rising, leveling off and then decreasing again with *d* value increasing. The *d* value in the leveling off stage is the best value for performance optimization. The best *d* value decreased with increasing MWCNT content of the shielding layer. An absorption dominated SE up to 65 dB and a high increment rate of 140% of SE were achieved by optimization of the *d* value and shielding layer.

## Abstract 2

Sandwich structure can be induced to achieve excellent electromagnetic interference shielding effectiveness (EMI SE) as is well known.

## Graphical abstract

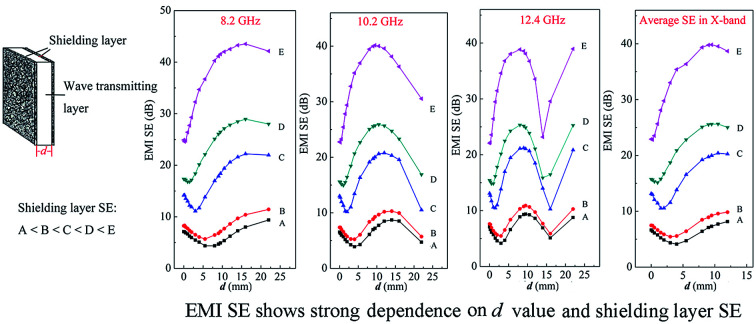

## 1 Introduction

Nowadays, the abuse of electronic device causes serious electromagnetic pollution, which has become one of the serious pollutions coming just after water pollution, air pollution and noise pollution. High-energy electromagnetic radiation may disturb the normal operation of electronic equipment, shorten the life of the equipment, result in the leakage of information and affect human health.^1–3^ Consequently, the development of materials that can act as microwave absorbers or shields against electromagnetic waves especially in the X-band has become essential.^4–6^ The electromagnetic interference shielding effectiveness (EMI SE) of shielding materials depends on the synergistic effect of EMI absorption, reflection and multiple reflection. Shielding performance can be enhanced by increasing the intrinsic EMI SE of shielding materials through improving electrical conductivity, magnetic permeability and thickness of shielding materials. However, designing a special structure is a simpler and more feasible way to improve shielding performance. Unfortunately, how to optimize the structure to achieve performance optimization is still a problem.

Metal sheets and resin-based metal micro-nanoparticle composites have been widely used to process into high-performance EMI shielding materials because of their excellent reflection property. But the application of metal materials is limited by their high density, poor processability and dispersibility.^2,3,7^ Moreover, secondary pollution caused by reflected electromagnetic waves is also a fatal limitation. Besides the conventional metal-based EMI shielding materials, resin matrix composites containing carbon-based nanofillers, particularly carbon nanotube (CNT)^8–10^ and graphene^11,12^ have become the focus of research due to their advantages of light weight, excellent processability, resistance to corrosion, and broad absorption bandwidth.^13–18^

CNTs are nano-scale hollow tubes with large L/D ratio. It has extraordinary electrical, thermal, mechanical properties and excellent absorbing properties. CNTs filled composites may be fit for new shielding materials with lightweight, broadband and high absorption efficiency. CNTs can be added directly into resin as fillers during preparation of composites.^19–22^ The compatibility between resin and CNTs can be improved by surface modification of CNTs to improve the dispersity at high filling levels.^23^ The lightweight CNT composites can be prepared by mechanical mixing easily, but the SE value of the composites is not high. Y. Huang, *et al.* prepared a type of single-walled carbon nanotube/epoxy composites of 2 mm thick showing an SE of only 20–30 dB with a high filler content of 15 wt%.^21^ Increasing the filling loading and its dispersity in resin is still key issue for obtaining a high SE value. In addition, for the applications in special fields, where the materials require not only excellent shielding performance but also superior thermal stability and excellent mechanical properties, high performance plastics rather than general plastics are demanded. Poly(phthalazinone etherketone) (PPEK) is a new type of amorphous polymer with the relatively high heat-resistance up to 400 °C and *T*_g_ higher than 250 °C.^24^ In addition, the twisty and non-coplanar structure of phthalazinone moieties of its molecular chain endows it a better solubility in chloroform and *N*-methyl-pyrrolidone (NMP) and some other organic solvents.^25,26^ In view of its superior performance, PPEK can be easily processed and applied in a variety of fields as high performance plastic and composite.

Taking the above factors into consideration, special structures have been induced to achieve excellent electromagnetic shielding performance, such as sandwich structure,^19,20,27–29^ gradient structure,^19,29^ porous structure^30,31^ and so on. The study of Jin Gyu Park, *et al.* showed that the composite laminated structures made with CNT bulkypaper (BP) and epoxy or polyethylene insulating layer can increase the EMI SE from 45 dB to close to 100 dB owing to the utilization of the double-shielding effect.^27^ Li Y., *et al.* introduced a wave-transmitting layer (pure polyurethane layer) between two pieces of polyurethane/graphene (PUG) foams to prepare a sandwich structure, by which the SE of the samples could be dramatically enhanced.^29^

Plastic matrix nanocomposite was prepared for the application in EMI shielding in many reports. Sandwich structure has been induced to achieve excellent EMI SE as reported in a few reports.^19,20,27–29^ However, how to optimize the structural to achieve performance optimization has not been thoroughly explored till date. Some investigation needs to be done to explore what factors affect the shielding property and how these factors affect shielding property. In this paper, PPEK and multiwall carbon nanotubes composite films (PPEK/MWCNT) were prepared as shielding layers with the aid of water induced phase separation (WIPS) process. A series of sandwich structure were designed by changing shielding layer and wave-transmitting layer. EMI SE dependence on the wave-transmitting layer thickness (*d*) and shielding layer SE was investigated in details.

## 2 Experimental

### 2.1 Materials

MWCNT (diameter: 10–20 nm, length: 10–30 μm) were procured from Chengdu Organic Chemicals CO., Ltd. Poly(phthalazinone etherketone) (PPEK) with a molecular weight of 70 000 was supplied by Dalian BaoLiMo New Materials Co., Ltd. PPEK powder was purified using chloroform. Anhydrous ethanol (AR), chloroform (AR) and 1-methyl-2-pyrrolidinone (NMP, AR) were purchased from LiaoDong Chemical Reagent (China).

### 2.2 Sample preparation

**Fig. 1:**
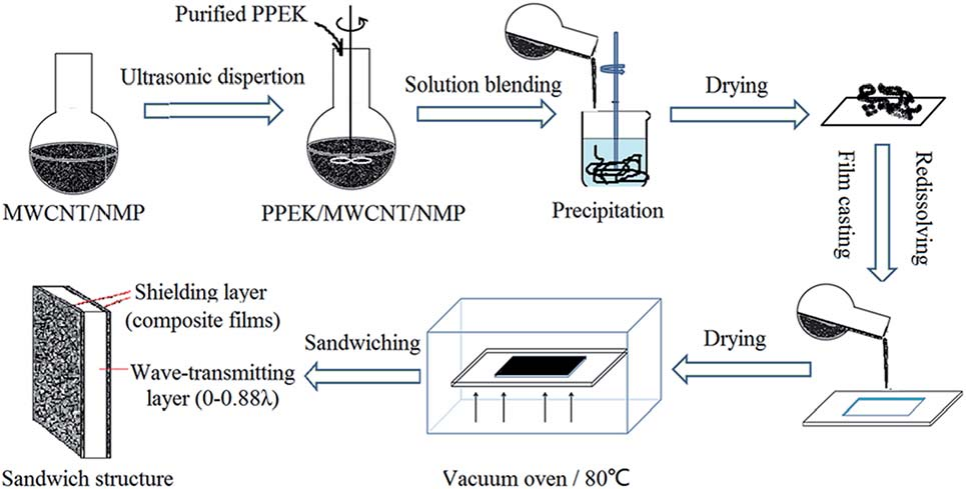
Preparation of composite film with WIPS method and sandwich structure of shielding materials.

The preparation procedure is illustrated in Fig. 1. At first PPEK/MWCNT composite films were prepared using WIPS technique. Then sandwich structure was designed by using two sheets of composite films as shielding layers and a pure PPEK resin sheet as wave-transmitting layer.

#### 2.2.1 Preparation of PPEK/MWCNT composite films

As a typical WIPS process, MWCNT (0.75 g) was ground for 30 minutes in a ball mill with several drops of NMP. The obtained slurry was transferred and dispersed in NMP followed by sonication for 0.5 h to get a homogeneous dispersion of 0.05 g mL^−1^. The MWCNT dispersion was added into a pre-dissolved PPEK solution (4.25 g PPEK) assuring that the content of MWCNT is 15%. The mixture was stirred mechanically for 2 h at 80 °C, and then treated by ultrasonic dispersion for 30 minutes at room temperature. The blend solution was precipitated with deionized water to form a strip blend following by filtrating and washing. After drying in a vacuum oven at 80 °C, the PPEK/MWCNT composite with 15 wt% loading of MWCNT was obtained and used as master batch.

The above PPEK/MWCNT (15 wt%) master batch was used for preparing PPEK/MWCNT composite film with 1–10 wt% CNT contents. A certain amount of PPEK/MWCNT master batch and purified PPEK were added to NMP solvent. By adjusting the mass ratio of PPEK/MWCNT master batch and PPEK the samples containing 1, 2, 3, 5, 10 and 15 wt% of MWCNTs were denoted as CNT1, CNT2, CNT3, CNT5, CNT10 and CNT15 respectively. The above mixtures were mechanically stirred at 80 °C for 2 h to obtain uniform mixing solutions. The solutions were casted on a self-made glass mold and dried at 80 °C in vacuum for 9 h to form composite films. The film thickness was designed as 100 or 200 μm. PPEK/MWCNT composite film with CNT loading of 5 wt% and thickness of 100 μm was named as CNT5-100. Others were named in the same way. The composite films are used as shielding layers to fabricating the sandwich structure in the following experiment.

#### 2.2.2 Preparation of sandwich structure

A sandwich structure of EMI shielding materials was designed by using two pieces of PPEK/MWCNT composite films and a pure resin sheet with different *d* value as shown in Fig. 1. Sandwich structure constructed by CNT5-100, CNT5-200, CNT10-100, CNT15-100 and CNT15-200 were denoted as sample A, B, C, D and E, respectively. The *d* value was set as 0 mm, 0.2 mm, 0.5 mm, 1 mm, 1.5 mm, 2 mm, 3 mm, 4 mm, 6 mm, 8 mm, 10 mm, 12 mm, 14 mm, 16 mm, and 22 mm corresponding to 0–0.88*λ* (*λ* is 25 mm, the minimum wavelength of the X-band).

### 2.3 Characterization

Cross-sectional morphology of composite film was observed by field emission scanning electron microscopy (NOVA Nano SEM 450). The fractured surfaces of samples were stuck onto the SEM holder followed by spraying gold for 30 s. The electrical resistance of composite films was measured by two-point probe method. Rectangular test samples (1 cm × 3 cm) were coated with a thin layer of conductive silver paste at the end, and then tested with HP4339B high resistance tester (*R* > 10^7^ Ω) and R6441A digital multimeter (*R* < 10^7^ Ω). The EMI shielding performance was characterized by waveguide measurement system using a vector network analyzer (3656D, The 41st Institute of China Electronics Technology Group Corporation) in the frequency range of 8.2–12.4 GHz (Fig. S1†). The samples were cut into a rectangular shape to fit in well with the chamber of the sample holder. The four main scattering parameters, S_11_, S_12_, S_21_ and S_22_ were detected.

The incident electromagnetic wave passing through a material undergoes four different processes: reflection, absorption, penetration and multiple reflection.^32,33^ The EMI shielding effectiveness (SE) should be the sum of its reflection energy and internal absorption energy. SE_R_, SE_A_ and EMI SE were analyzed by the following equations^23,34,35^ based on the values of *S*_11_, *S*_12_, *S*_21_ and *S*_22_:

1 *R* = |*S*_11_|^2^ = |*S*_22_|^2^

2 *T* = |*S*_12_|^2^ = |*S*_21_|^2^

3 *A* = 1 − *R* − *T*

4 SE_R_ = 10 log(1/(1 − *R*))

5 SE_A_ = 10 log((1 − *R*)/*T*)

6 SE = SE_A_ + SE_R_

where, *R*, *T* and *A* present the reflection coefficient, transmission coefficient, and absorption coefficient, respectively.

## 3 Results and discussion

### 3.1 Morphology and electrical properties of PPEK/MWCNT composite films

**Fig. 2:**
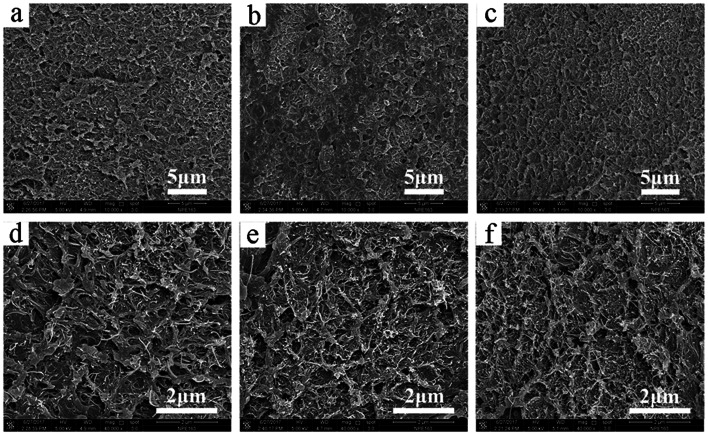
SEM micrographs of the fractured surfaces of PPEK/MWCNT composite films with MWCNT loading of: (a and d) 5 wt%, (b and e) 10 wt% and (c and f) 15 wt%.

The morphology of PPEK/MWCNT composite films is showed in Fig. 2. MWCNTs can be uniformly dispersed in composite even at a high loading of 15 wt%. In addition, MWCNTs are overlapped with each other to form a conductive path. The WIPS method has a great advantage over the conventional melt-compounding method for making good filler dispersion especially at high filler loading. Generally, high loading of nanofillers in polymer matrix during compounding process tends to give an obvious increase in melt viscosity due to the extremely high specific surface area of nanofillers. In the present experiment, at first a large amount of MWCNTs were added into PPEK solution at a low concentration and then the PPEK/MWCNT nanocomposite was obtained by precipitation through the WIPS process. This WIPS method can effectively avoid serious processing problems caused by high viscosity of the mixture.

**Fig. 3:**
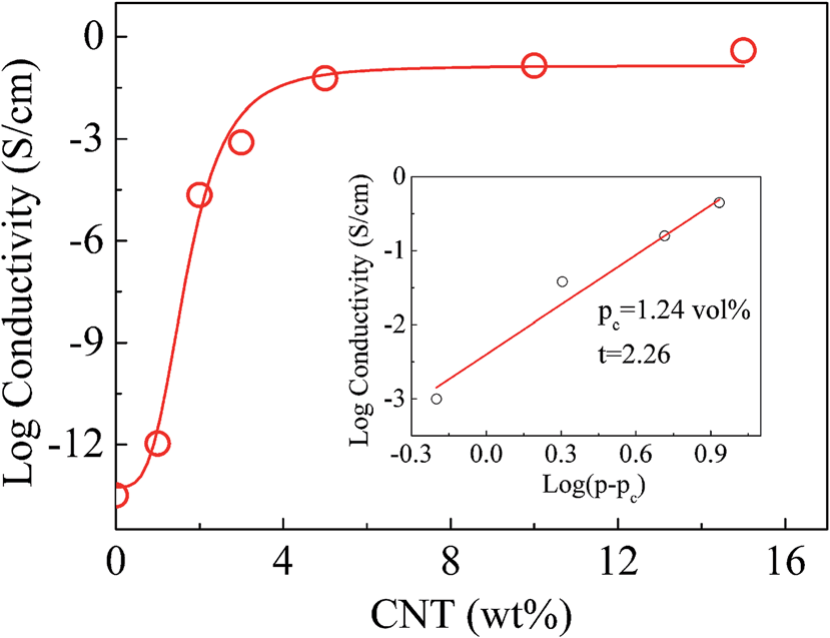
The logarithm of electrical conductivity of PPEK/MWCNT composite films with different MWCNT loading. The inset is the log–log plot of conductivity *versus* (*p* − *p*_c_) and the solid line is a fit to the measured data using power-law equation, *i.e. σ* = *σ*_0_(*p* − *p*_c_)^*t*^.

The EMI shielding performance of conductive composites is closely related to their electrical properties.^27,36–38^ The electrical conductivity of PPEK/MWCNT composite films was studied. Fig. 3 shows the nonlinear relationship between the logarithms of electrical conductivity of PPEK/MWCNT composite films and MWCNT mass fraction. The conductivity of composite film with 1 wt% loading of MWCNT was 10^−12^ S cm^−1^ level. Then the electrical conductivity reached 6 × 10^−4^ S cm^−1^ when the content of MWCNT increased to 2 wt%, indicating the formation of conductive networks and up to the percolation threshold. As further increase in MWCNT loading, the growth trend of conductivity became gentle. On the whole, the conductivity of the PPEK/MWCNT composite films displays a great promotion over 13 orders of magnitude as MWCNT content higher than 5 wt% and reached 0.39 S cm^−1^ at 15 wt% of MWCNTs. As shown in the inset of Fig. 3, the relationship of the electrical conductivity and the MWCNT loading of PPEK/MWCNT composites was evaluated with power-law equation,^4,39^*i.e. σ* = *σ*_0_(*p* − *p*_c_)^*t*^. In the formula, *σ* represents the electrical conductivity of PPEK/MWCNT composites, *σ*_0_ is a constant related to conductivity of fillers, *p* is the volume fraction of fillers, and *p*_c_ is the percolation threshold. *t* value can be used to judge the dimensionality of the conductive system. The low *p*_c_ of 1.24 vol% results from the uniform dispersion of MWCNTs, and the obtained *t* is 2.26, confirming the formation of 3D conductive networks. The increase of MWCNT content also makes for improving thermal stability (Fig. S2 and Table S1; ESI†) and mechanical property (Fig. S3; ESI†) of composite films.

### 3.2 EMI shielding performance of composite film and its sandwich structure in X-band

#### 3.2.1 EMI SE of PPEK/MWCNT composite films

**Fig. 4:**
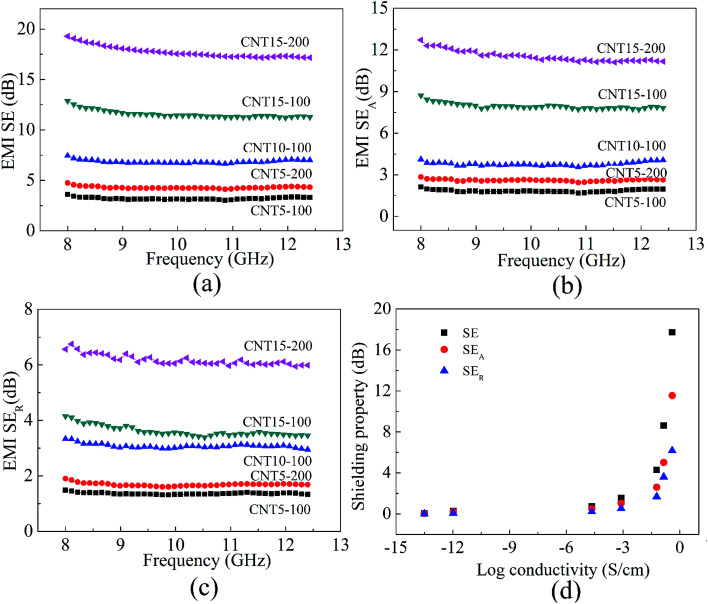
(a) EMI SE, (b) SE_A_ and (c) SE_R_*versus* frequency for PPEK/MWCNT composite films in X-band; (d) variation in the value of SE, SE_A_ and SE_R_ with electrical conductivity of PPEK/MWCNT composite films (200 μm).

The EMI shielding performance of PPEK/MWCNT composite films with MWCNT content 5, 10 and 15 wt% and different thickness was detected in X-band. The corresponding SE, absorption loss (SE_A_), reflection loss (SE_R_) and their dependence on electrical conductivity were shown in Fig. 4.

The low electrical conductivity of PPEK/MWCNT composite films with filler loading less than 3 wt% induced almost no EMI shielding which was not shown here. As increasing filler content, the EMI SE of CNT5-100 is about 3 dB, and CNT15-100 increases to 12 dB (>90% attenuation). CNT15-200 reveals a shielding effectiveness of nearly 20 dB, and the SE is almost independent of frequency change in the X-band.

For further investigation of the shielding mechanism, SE for PPEK/MWCNT composites is resolved into resultant SE_A_ and SE_R_ components as shown in Fig. 4b and c, respectively. The result shows that SE_R_ increases with increase in MWCNTs loading because of increase in electrical conductivity of the composites (Fig. 4d). However, with increasing of MWCNT content, SE_A_ increases more rapidly compared to corresponding SE_R_ component. Such a behavior is direct consequence of square root and logarithmic dependence of SE_A_ and SE_R_ on conductivity respectively.^40,41^

When the content of MWCNT is more than 15 wt%, it is difficult for MWCNT to disperse in PPEK uniformly due to plenty of entanglements, thus the improvement of EMI shielding performance is limited. In this case, increasing the thickness of shielding materials or designing special structure is a more effective way to enhance the shielding performance.

#### 3.2.2 EMI SE of sandwiched PPEK/MWCNT composite films

In order to promote EMI SE, the above composite films with different SE were used as shielding layers to fabricate sandwich structures by introducing a pure resin sheet between them. The influence of *d* value and the intrinsic SE of shielding layers on the EMI SE of sandwich structure were investigated in details. Here we introduce a parameter *G*, the increment rate of EMI SE value. When the *d* value is 0, the EMI SE was defined as the corresponding initial value (SE_0_). The increment rate *G* of EMI SE refers to the increment of SE divided by SE_0_ as shown in eqn (7), where the increment of SE is an average value that subtracting the SE_0_ from maximum SE (SE_max_) in 8.2–12.4 GHz.

7 *G* = (SE_max_ − SE_0_)/SE_0_ × 100%

**Fig. 5:**
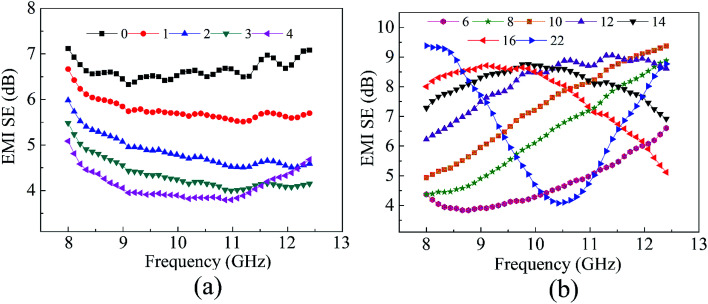
The plot of SE for the sample A with different *d* values in the frequency of 8.2–12.4 GHz. The annotations in the figure represent the *d* and the unit is millimeter.

Fig. 5 shows the EMI SE of sample A with two shield layers (CNT5-100) and a wave-transmitting resin sheet (*d* = 0–22 mm). While *d* < 4 mm, the SE decreases as *d* value increasing. But further increasing in *d* value, EMI SE increased. Even though, the maximum SE is less than 10 dB which is too low to meet the request for commercial utilization. The resonance peak where shielding performance descended dramatically appeared while *d* > 0.56*λ* (14 mm). The resonance peak appeared at *f* > 12.40 GHz (*d* = 16 mm, 0.64*λ*) and *f* = 10.45 GHz (*d* = 22 mm, 0.88*λ*).

**Fig. 6:**
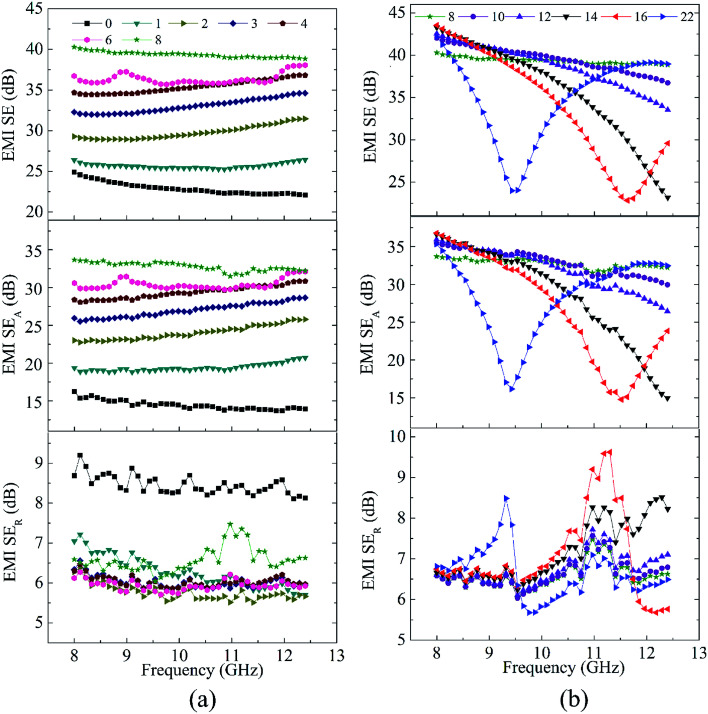
The SE, SE_A_ and SE_R_ of sample E with different *d* value in X-band. The annotations in the figure represent the *d* value and the unit is millimeters.

The same experiment and analysis were carried out for other four series of samples B, C, D and E. To shorten the paper, the corresponding detailed results (SE, *A*, *R*, *etc.*) were shown as Fig. S4–S7 in ESI† respectively. The SE test result of sample E was shown in Fig. 6.

Here in, we take sample E (Fig. 6 and S7, ESI†) to systematically illuminate the influence of wave-transmitting layer on shielding properties of sandwich structure by analyzing its SE, *A*, *R*, *etc.*

The SE of sample E increases monotonically with increasing of the *d* (Fig. 6a). And the increase in SE stopped at 0.32*λ* (8 mm) as a result of the limitation of SE_R_ and multiple SE_R_.^27^ Sample E can achieve an initial SE_A_ of 16 dB (average value) while a SE_R_ of only 8.5 dB. When the SE_A_ reached as high as 33 dB (*d* = 0.32*λ*, 8 mm), the SE_R_ still remained only about 6.6 dB. Thus the shielding material exhibits an improved SE coupled with an absorption dominating shielding mechanism, which is different from the previously reported reflection dominated polymer/graphene and polymer/CNT nanocomposites.^38,42,43^ Moreover, the obtained SE (*ca.* 40 dB) is greater than three-layered PUG composites with higher graphene loading (16 wt%) and much thicker shielding layer (2.4 mm).^29^ Sandwich structure of graphene paper/wax/graphene paper^28^ also presents a lower SE in most frequency bands than sample E due to its thin wax layer. The amplitude of the reflected electromagnetic waves depends on impedance matching degree between the shielding material and resin. CNT15-200 is a good conductive material with much lower impedance than resin, so it shows a higher impedance mismatch and greater reflection coefficient *R* (Fig. S7e†). The incident microwaves can be reflected and scattered many times at the interface between MWCNT and resin, and the microwaves were difficult to escape from the system until being absorbed and converted into heat.^44,45^ Thus the electromagnetic waves can be attenuated in form of absorption effectively, while the corresponding SE_R_ is greatly suppressed because of the constructive interference.^28^ The results are consistent with those in Fig. S6,† and can be demonstrated by the decrease of reflection coefficient *R* and increase of absorption coefficient A (Fig. S6e and S7e, ESI†).

As further increase in *d* value (*d* > 0.4*λ*, 10 mm), resonance peak appeared in X-band range. This phenomenon indicates that the increase of SE caused by increasing the *d* value is contributed by the constructive interference.^28,29^ In a typical Fabry–Pérot cavity, multiple reflection waves can be aligned between parallel reflecting planes, producing constructive interference when the reflected waves are in phase.^28^ Resonance peak is observed when the *d* does not match well with the distance for the occurrence of constructive interference. This phenomenon results in a decrease of absorption coefficient A and an increase of transmission coefficient *T* at the same time (Fig. S7f, ESI†). It gives a good explanation to the dramatic descending of SE, SE_A_ and the slight increase of SE_R_.

**Fig. 7:**
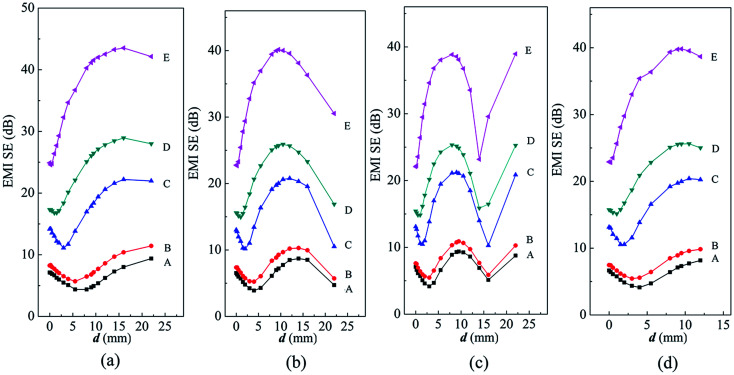
Dependence of EMI SE on *d* value of sandwiched samples at frequency of: (a) 8.2 GHz, (b) 10.2 GHz and (c) 12.4 GHz; (d) average SE before the appearance of resonance peak in X-band.

**Fig. 8:**
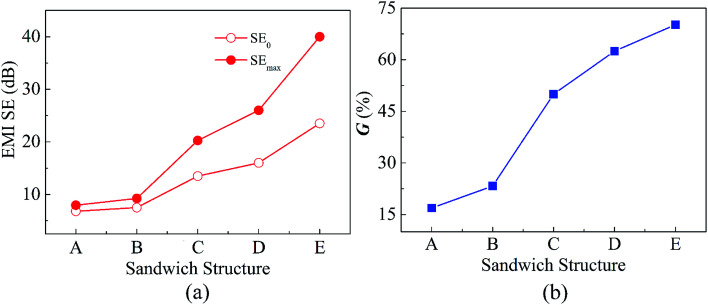
(a) Initial and maximum EMI SE, SE_0_ and SE_max_, of sample A, B, C, D and E; (b) the increment rate *G* of EMI SE.

There is a strong correlation between the SE of sandwich structure and shielding layer. To achieve desired shielding level, some investigation needs to be done to find out and alleviate the constraints. After analyzing the Fig. 5 and S4–S7 (ESI†), the *d* value dependence of the EMI SE was described in Fig. 7 for these sandwich samples. The SE at 8.2 GHz, 10.2 GHz and 12.4 GHz, and average SE in X-band before the appearance of resonance peak were presented in Fig. 7 respectively. In addition, the SE_0_, SE_max_ and the increment rate *G* of EMI SE were shown in Fig. 8. The center frequency of resonance peaks of these sandwich structures were listed in Table S2 (ESI†).

As shown in Fig. 7, the EMI SE of five samples show a tendency as E > D > C > B > A with the same *d* value at the same frequency. This is closely related to the filler content and thickness of shielding layer.

The SE of sample A, B, C and D shows the same tendency with the variation of *d* value. With the *d* increasing, the SE decreased firstly (decrease stage), then showed an upward tendency (rising stage) until reaching a plateau, and finally descended (re-decreasing stage) at some frequency band. As explained in Fig. 7, in the first stage the SE decreased resulting from the mismatch between the *d* and the distance of forming constructive interference. The *d* value range in the first stage of sample B (*d* < 4 mm), C (*d* < 2 mm) and D (*d* < 1.5 mm) decreases in turn. There is no descending in SE for sample E even at very small *d* value.

In the second stage, the EMI SE increased as increasing of *d* value. SE reached the maximum value when the *d* values are 0.56*λ* (14 mm), 0.48*λ* (12 mm), 0.4*λ* (10 mm), 0.32*λ* (8 mm) and 0.32*λ* (8 mm) for sample A, B, C, D and E respectively. It is interesting to note that the difference between *d* values is approximately 0.32*λ* for achieving the maximum and the lowest SE for each sandwich structure. Fig. 8 shows SE_0_, SE_max_ and the *G* for five series of samples. The *G* for sample E was up to 70.2% and the SE_max_ reached to 42 dB. However the SE of sample A, B, C and D is still lower than the commercial level of 30 dB even though introducing a large wave-transmitting layer. Clearly, a higher intrinsic SE of shielding layer makes for decreasing optimal *d* value and increasing the increment rate *G* of the sandwich structure.

With further increase in *d* value, SE decreased due to that the resonance peak appears. Table S2 (ESI†) shows that the resonance peak shifted to lower frequency at larger *d*, which is consistent with the phenomenon reported by Li Y.^29^ The shifting is due to that longer wavelengths are required by the constructive interference occurring in larger distances. Fig. S8 (ESI†) depicts the dependence of EMI SE on frequency of sandwiched samples at specific *d* values, 0.56*λ*, 0.64*λ* and 0.88*λ*. The resonance peak goes to lower frequency with the intrinsic SE of shielding layer increasing at specific *d* value. Moreover, the width of resonance peak becomes narrower as shown in Fig. S8c (ESI†).

Afterwards, three pieces of shielding layers were used for preparing a sandwiched shielding material with or without wave-transmitting layer to further investigate the influence of wave-transmitting layer on the SE. Denotation of sandwiched and triple-layer composites are shown in Table S3 (ESI†). Sample F is a sample prepared by sandwiching two wave-transmitting layers of 11 mm thick among three pieces of CNT15-200. A triple-layered composite, sample G, was prepared by stacking three pieces of CNT15-200 face to face without wave-transmitting layer.

**Fig. 9:**
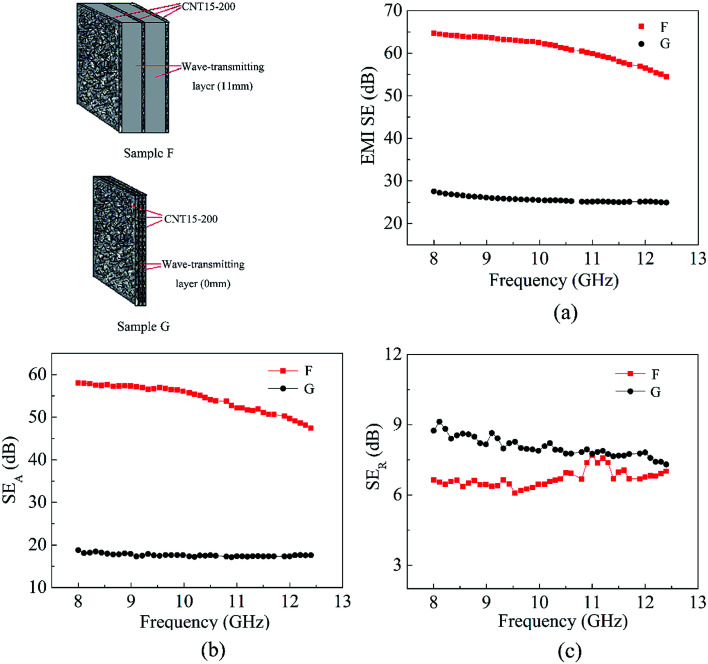
Plots of (a) EMI SE, (b) SE_A_ and (c) SE_R_*versus* frequency for sample G and sample F in the X-band.

The EMI shielding performance of sample F and G are shown in Fig. 9. Sample F has an average SE of 61.5 dB in frequency range of 8.2–12.4 GHz which is much higher than that of sample G (25.7 dB). Furthermore, the improvement of the SE mainly ascribed to the increase of SE_A_ (Fig. 9b). Given from the appropriate thickness of wave-transmitting layer, the SE of sample F is even higher than sandwich structure of BP/PE layer.^27^ Notably, the SE of the sandwich structure with two wave-transmitting layers (sample F) increased by 140% compared to that of multilayer structure (triple-layered sample G) with the same components of PPEK/MWCNT composite films. By contrast, the SE of sandwiched shielding material with one wave-transmitting layer (sample E) increases only by 70.2% compared to corresponding SE_0_ (Fig. 8). Based on this, we speculate that increasing wave-transmitting layer with best *d* value is beneficial to increase the increment rate of SE.

## 4 Conclusion

PPEK/MWCNT composite films with homodisperse of MWCNT were prepared with the aid of WIPS process. The EMI shielding performance of composite films was evaluated in X-band. Sandwich structures were designed by sandwiching a pure resin sheet as a wave-transmitting layer to systematically investigate the influence of wave-transmitting layer and shielding layer on shielding properties by comparing their SE, *A*, *R*, *etc.* With the *d* increasing, the SE of sandwich structure decreased firstly, then upward reached a plateau, and then descended at some frequency band corresponding to the appearance of resonance peaks. The intrinsic SE of the shielding layer was negatively correlative to the best *d* value, while positively correlative to the increment rate *G* of the SE. However, it is interesting to note that the *d* value interval of the rising stage is almost constant, *ca.* 0.32*λ*. An absorption dominated SE up to 65 dB and high increment rate of 140% can be achieved by optimization of *d* value and shielding layer.

## Conflicts of interest

There are no conflicts of interest to declare.

## Supplementary Material

RA-009-C9RA02959A-s001
