# Supplementary material for: High absorption shielding material of poly(phthalazinone etherketone)/multiwall carbon nanotube composite films with sandwich configurations
Source: RSC Adv. 2019 Jun 14;9(33):18758–66. doi: 10.1039/c9ra02959a (PMC9064811; doi:10.1039/c9ra02959a)
Supplement: RA-009-C9RA02959A-s001 [file RA-009-C9RA02959A-s001.pdf]

Electronic Supplementary Material (ESI) for RSC Advances.
This journal is © The Royal Society of Chemistry 2019

Supporting Information

High Absorption Shielding Material of Poly(phthalazinone etherketone)/Multiwall Carbon Nanotube Composite Films with Sandwich Configurations

Yunping Hu [a], Ping Tang [a], Longwei Li [a], Junyu Yang [b], Xigao Jian [a]， Yuezhen Bin [a*]

[a] Department of Polymer Science and Engineering, Faculty of Chemical, Environmental and Biological Science and Technology, Dalian University of Technology, No.2 Linggong Rd, Dalian, 116024, P. R. China

[b] Department of Chemical Engineering, University of Pittsburgh, 4200 Fifth Avenue, Pittsburgh, PA 15260, USA

[*]Corresponding author.

Tel: +86- 411- 84986093.

E-mail address: binyz@dlut.edu.cn (0000-0002-3188-721X)

Supplementary information:

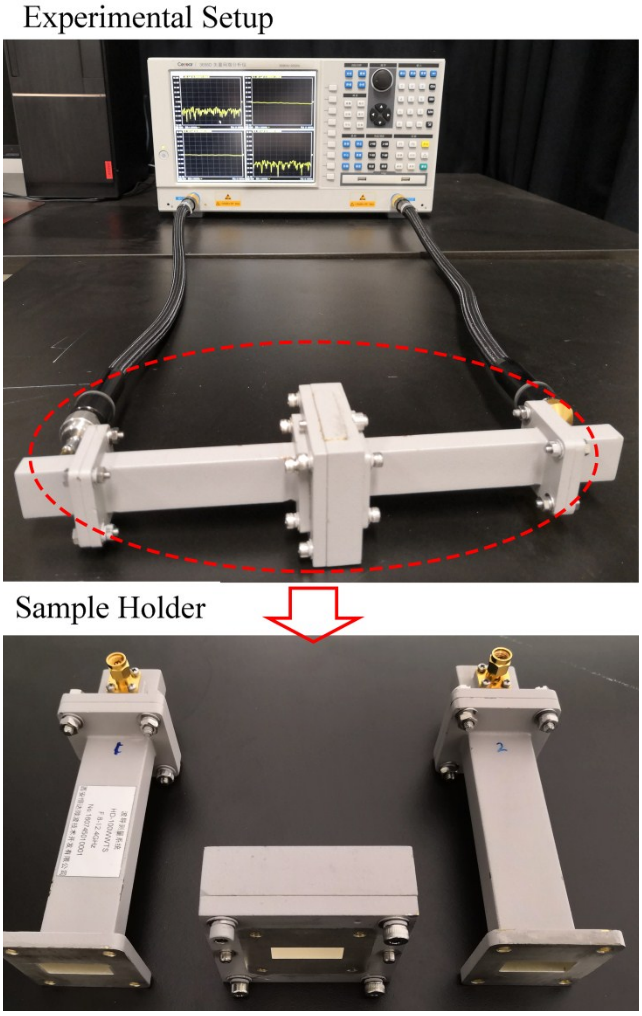

Fig. S1 Digital pictures of experimental setup and the sample holder for testing of shielding property.

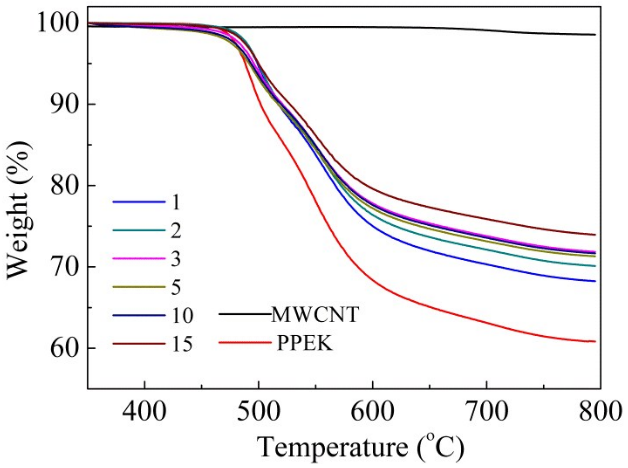

Fig. S2 TG traces of MWCNT, PPEK, and PPEK/MWCNT composites with different mass ratio of MWCNT.

Table S1 Results of TG traces for PEK and PEK/CNF composites:

MWCNT loading (wt%)	T_{max}^{a} (°C)	Char residue (%) at 800 °C
0 (PPEK)	496.6	60.83
1	496.5	68.24
2	498.7	70.11
5	498.7	71.85
10	502.3	71.3
15	502.8	71.67
100% (MWCNT)	501.4	73.95

[a] Temperature at which rate of mass loss is maximum

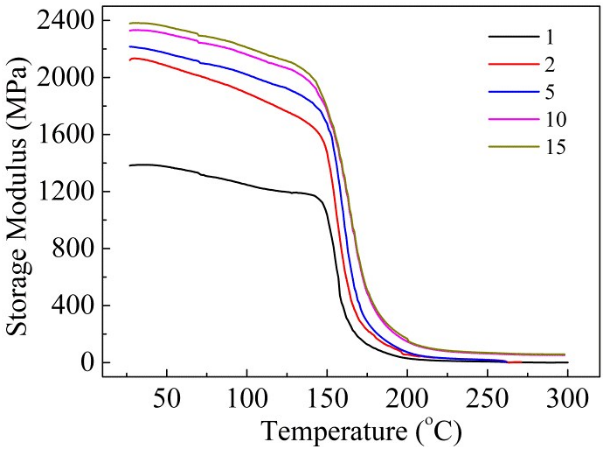

Fig. S3 The storage modulus of PPEK/MWCNT composites with different mass ratio of MWCNT.

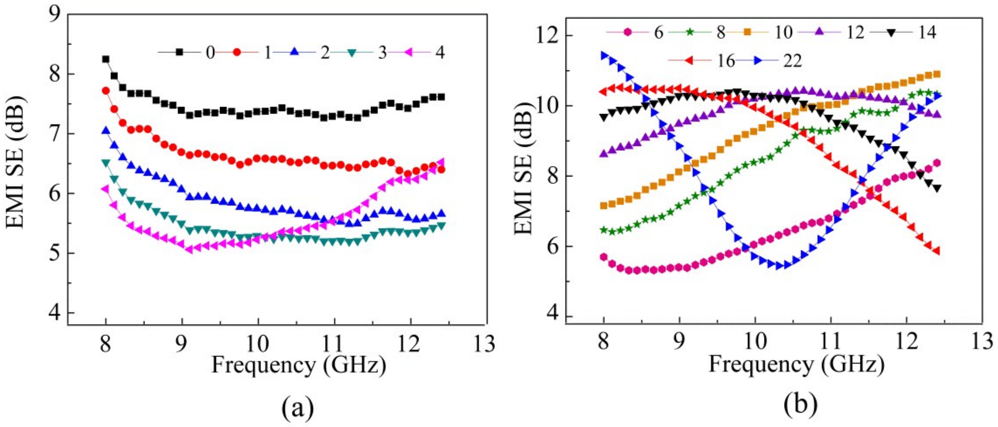

Fig. S4 The SE of sample B with different ***d*** value in the frequency of 8.2–12.4 GHz, the annotations in the figure represent the ***d*** and the unit is millimeter.

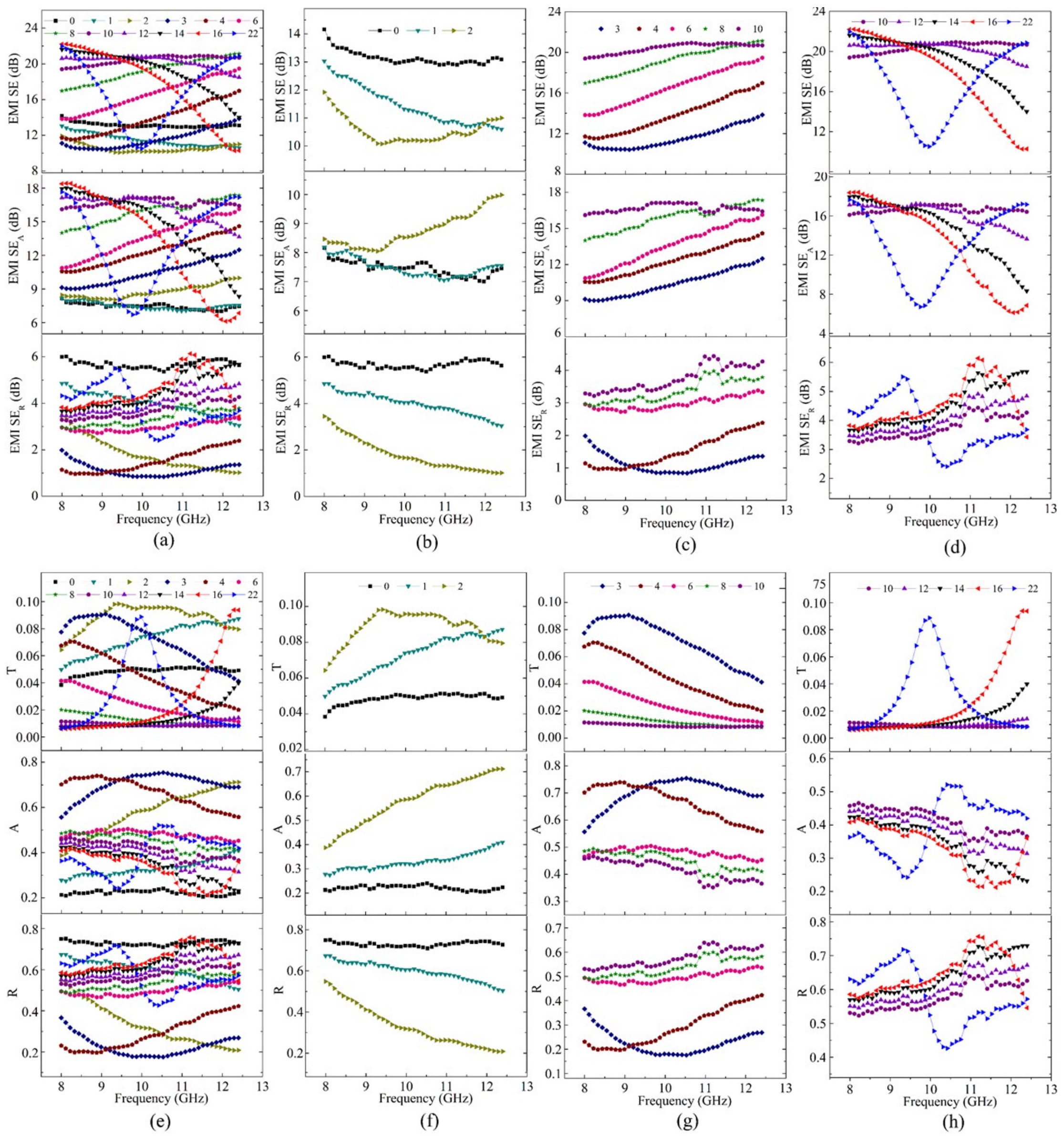

Fig. S5 a-d) the SE, SE_A and SE_R of sample C in X-band with different ***d*** value: a) 0-22 mm, b) 0-2 mm, c) 3-10 mm and d) 10-22 mm; e-f) the T, A and R of sample C with different ***d*** value: e) 0-22mm, f) 0-2mm, g)3-10 mm and h) 10-22mm, the annotations in the figure represent the ***d*** and the unit is millimeter.

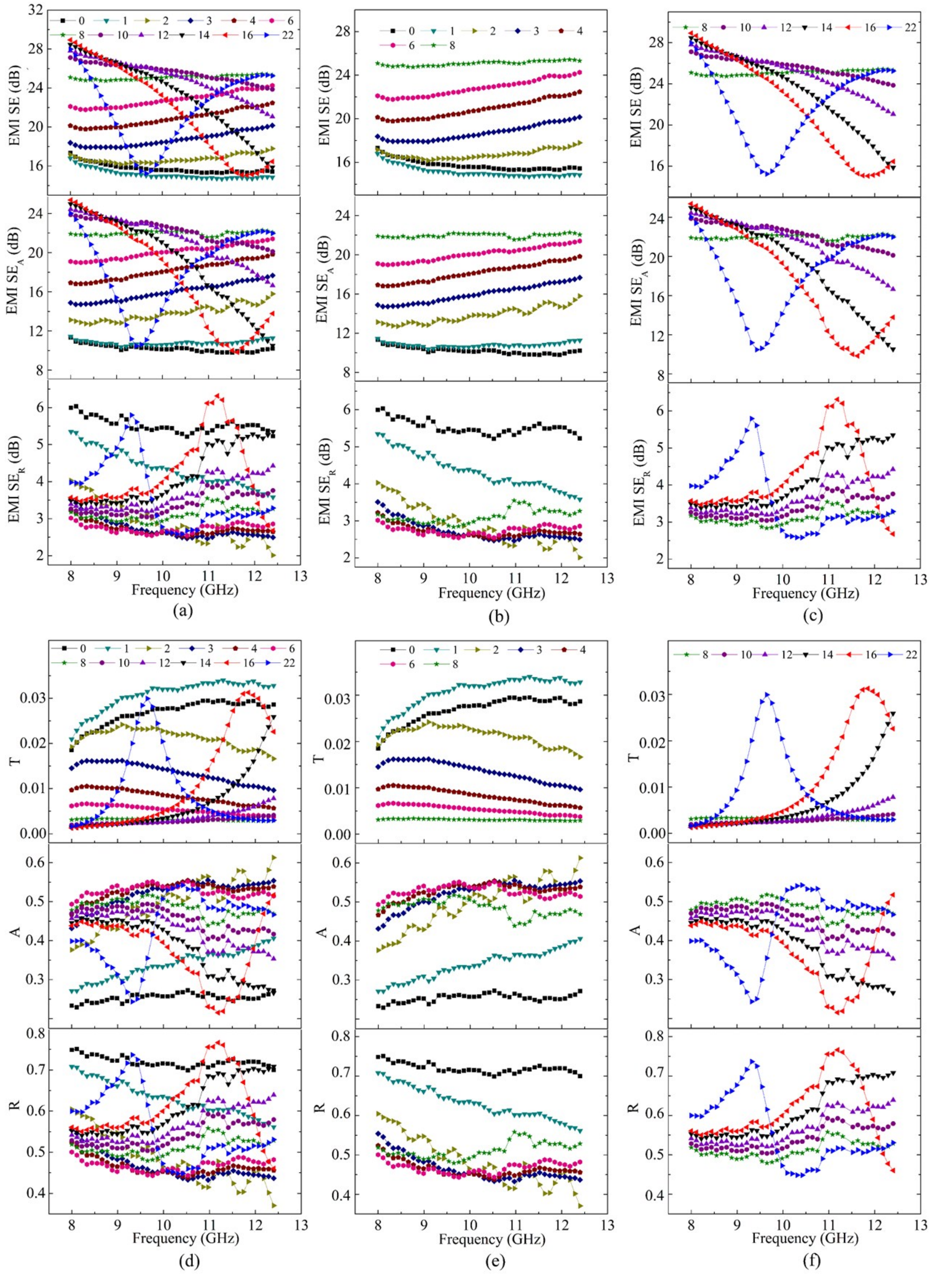

Fig. S6 a-c) the SE, SE$_A$ and SE$_R$ of sample D in X-band with different ***d*** value: a) 0-22 mm, b) 0-8 mm and c) 8-22 mm; d-f) the T, A and R of sample D with different ***d*** value: e) 0-22mm, f) 0-8mm and g) 8-22 mm, the annotations in the figure represent the ***d*** and the unit is millimeter.

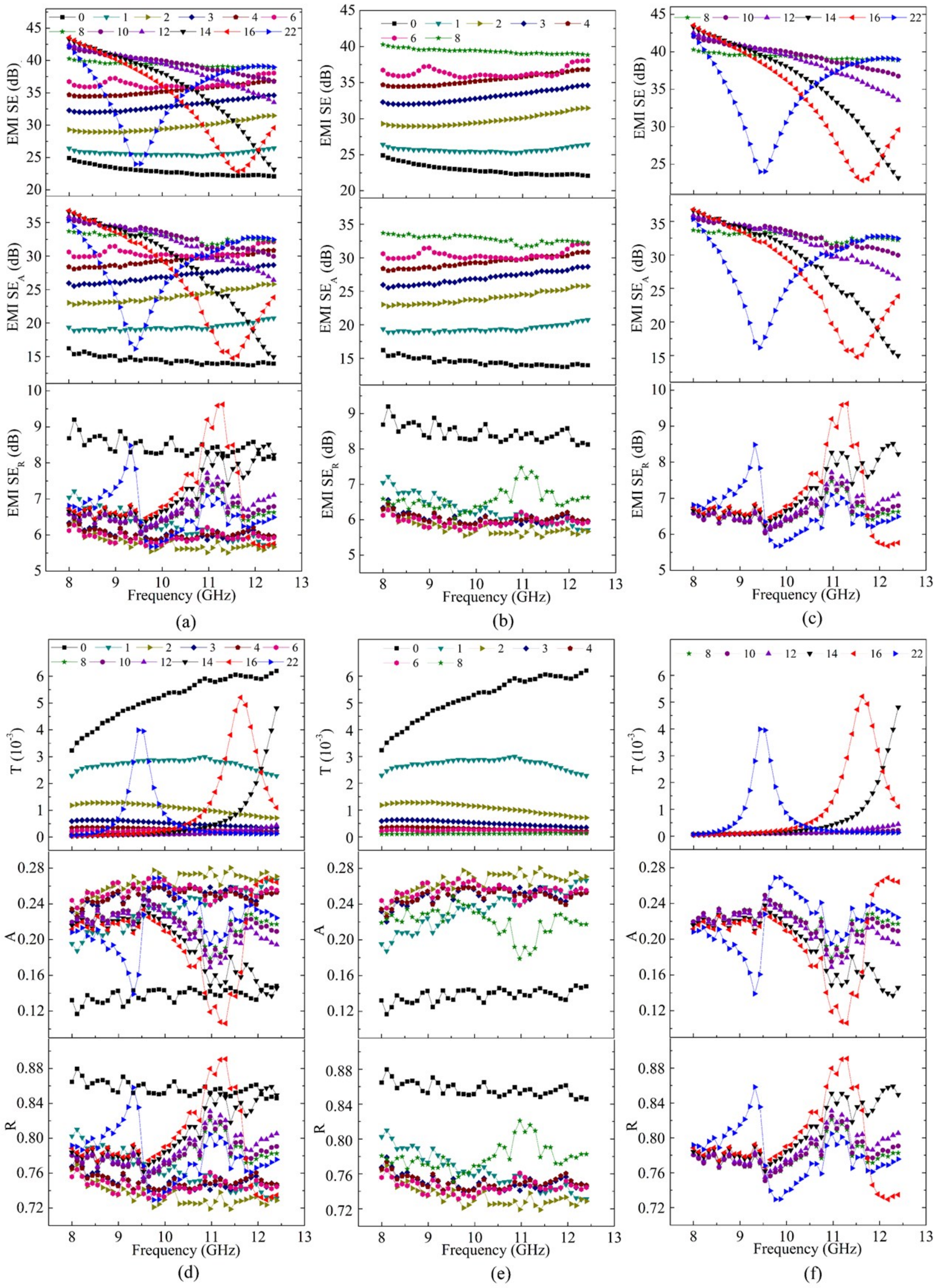

Fig. S7 a-c) the SE, SE_A and SE_R of sample E in X-band with different ***d*** value: a) 0-22 mm, b) 0-8 mm and c) 8-22 mm; d-f) the T, A and R of sample E with different ***d*** value: e) 0-22mm, f) 0-8mm and g) 8-22 mm, the annotations in the figure represent the ***d*** and the unit is millimeter.

Table S2 The position of resonance peak of samples with sandwich structure

d [λ]	Frequency [GHz]				
	A	B	C	D	E
**0.56**	-	>12.40	>12.40	>12.40	>12.40
**0.64**	>12.40	>12.40	12.39	11.88	11.62
**0.88**	10.45	10.32	9.96	9.65	9.46

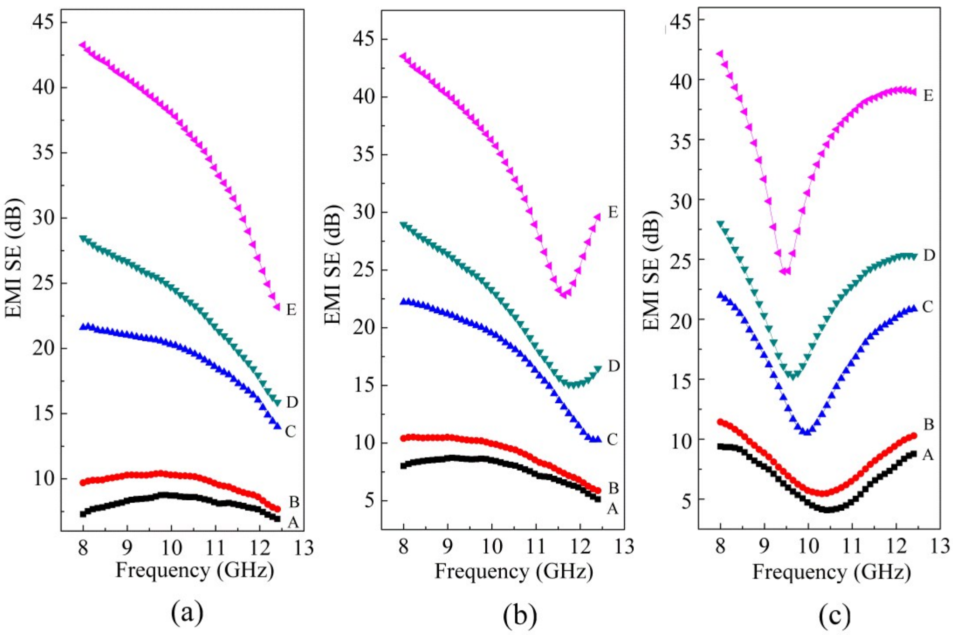

Fig. S8 Dependence of EMI SE on frequency of sandwiched samples at specific ***d*** values: a) 0.56λ, i.e., 14mm, b) 0.64λ, i.e., 16mm and c) 0.88λ, i.e., 22mm.

Table S3 List of sandwiched and triple-layered sample.

Sample	Number of Shielding Layer	***d*** (mm)	***d*** (λ)
E	2	8	0.32
F	3	11,11	0.44, 0.44
G	3	0,0	0, 0

Notes:

EMI — electromagnetic interference

SE — shielding effectiveness

SE_A — absorption loss

SE_R — reflection loss

T — transmission coefficient

A — absorption coefficient

R — reflection coefficient

d — the thickness of wave-transmitting layer

λ — λ is 25mm, the minimum wavelength of the X-band

PPEK — Poly(phthalazinone etherketone)

MWCNT — multiwall carbon nanotube

WIPS — water induced phase separation

NMP — N-methyl pyrrolidinone
